# Clinical Features and Treatment Outcomes of Medication Overuse Headache in Older Patients: Insights from a Nationwide Prospective Registry

**DOI:** 10.3390/jcm14144948

**Published:** 2025-07-12

**Authors:** Yooha Hong, Mi-Kyoung Kang, Hong-Kyun Park, Min Kyung Chu, Sun-Young Oh, Jin-Ju Kang, Heui-Soo Moon, Mi Ji Lee, Tae-Jin Song, on behalf of the RELEASE Investigators

**Affiliations:** 1 Department of Neurology, Dongtan Sacred Heart Hospital, Hallym University College of Medicine, Hwaseong 18450, Republic of Korea; dbgk486@naver.com (Y.H.); twomk4960@hallym.or.kr (M.-K.K.); 2 Department of Neurology, Inje University Ilsan Paik Hospital, Inje University College of Medicine, Goyang 10380, Republic of Korea; hkholes@gmail.com; 3 Department of Neurology, Severance Hospital, Yonsei University College of Medicine, Seoul 03722, Republic of Korea; chumk@yonsei.ac.kr; 4 Department of Neurology, Jeonbuk National University Hospital, Jeonbuk National University School of Medicine, Jeonju 54896, Republic of Korea; ohsun@jbnu.ac.kr (S.-Y.O.); jinj_k@naver.com (J.-J.K.); 5 Department of Neurology, Kangbuk Samsung Hospital, Sungkyunkwan University School of Medicine, Seoul 03181, Republic of Korea; drmhs0801@gmail.com; 6 Department of Neurology, Seoul National University Hospital, Seoul National University College of Medicine, Seoul 03080, Republic of Korea; mijilee.md@snu.ac.kr; 7 Department of Neurology, Seoul Hospital, Ewha Womans University College of Medicine, 260 Gonghang-daero, Gangseo-gu, Seoul 07804, Republic of Korea; 8 The Korean Headache Society, Hangeulbiseok-ro, Nowon-gu, Seoul 01830, Republic of Korea; kheadache2014@gmail.com

**Keywords:** medication overuse headache, older adult, preventive treatment, adherence, headache disability, aging

## Abstract

**Background and Objectives:** Medication overuse headache (MOH) presents unique clinical challenges in older adults due to age-related changes and comorbidities. However, data on MOH characteristics and treatment responses in this population remain limited. This study investigated the clinical features, treatment patterns, and short-term outcomes of MOH in older patients. **Methods:** We analyzed data from the RELEASE registry, a nationwide, multicenter prospective cohort of MOH patients in South Korea. Participants were stratified into older (≥65 years) and younger (<65 years) groups. We compared clinical features, treatment patterns, and 3-month outcomes, and identified factors associated with treatment response in the older group. **Results:** Among 791 patients, 72 (9.1%) were older. Compared to younger patients, older patients reported more monthly headache days (30.0 vs. 27.0, *p* = 0.012), more days using acute medication (30.0 vs. 20.0, *p* < 0.001), and fewer headache-free days (0.0 vs. 3.0, *p* = 0.012). They also experienced more severe headache days (12.5 vs. 10.0, *p* = 0.056). Despite this, older patients showed lower disability, with significantly lower Migraine Disability Assessment scores (30.0 vs. 46.0, *p* < 0.001) and a trend toward lower Headache Impact Test-6 scores (64.5 vs. 66.0, *p* = 0.065). In multivariable analysis, poor adherence to preventive treatment (≤24%) was significantly associated with non-response (OR 0.13, 95% CI: 0.02–0.96, *p* = 0.045) at 3 months. **Conclusions:** Older patients with MOH showed distinct clinical features, including higher headache frequency and severity but relatively lower disability. Improving adherence to preventive treatment may enhance treatment response. Age-specific management strategies are needed.

## 1. Introduction

Medication overuse headache (MOH) is a prevalent and disabling chronic disorder, affecting approximately 1–2% of the general population [1,2]. It places a considerable burden on both individuals and healthcare systems, contributing to reduced quality of life and increased healthcare utilization [3,4]. Although MOH is classified as a secondary headache disorder, it commonly arises in individuals with pre-existing primary headache syndromes, particularly migraine [5]. The high prevalence and associated disability of MOH contribute to significant socioeconomic impact across diverse populations [6,7]. As such, effective MOH management requires coordinated strategies emphasizing prevention and personalized treatment approaches [8].

In older adults, headache disorders pose distinct clinical challenges. This population may present with atypical headache phenotypes, including hypnic headaches, typical aura without headache, and secondary headache disorders such as MOH. Age-related physiological changes—such as altered pharmacokinetics, multiple comorbidities, and increased vulnerability to adverse drug reactions—further complicate both diagnosis and treatment in this population [9,10,11]. Despite representing a growing proportion of patients with headache disorder [12], older patients remain underrepresented in MOH research, with limited data on their clinical characteristics, treatment patterns, and outcomes.

Given the unique physiological and therapeutic context of aging, further investigation is warranted to understand the presentation and management of MOH in older patients. This study, based on data from a nationwide, multicenter prospective registry, aimed to characterize the demographic, clinical, and therapeutic features of older patients with MOH compared to younger individuals. We also sought to identify factors associated with treatment response in older adults, to inform age-specific management strategies for this underserved group.

## 2. Materials and Methods

### 2.1. Study Design and Participants

This study utilized data from the Registry for Load and Management of Medication Overuse Headache (RELEASE), a nationwide, multicenter, prospective observational study conducted in South Korea. A total of 791 patients diagnosed with MOH were consecutively enrolled from seven tertiary hospitals with specialized headache clinics between 1 April 2020 and 31 December 2024. A total of 791 patients diagnosed with MOH were consecutively enrolled between 1 April 2020, and 31 December 2024, from seven tertiary hospitals with specialized headache clinics [13]. The study protocol was reviewed and approved by the Institutional Review Board of Dongtan Hallym University Sacred Heart Hospital, Republic of Korea (approval number: Dongtan 2020-02-004), and all study procedures adhered to the principles of the Declaration of Helsinki.

Eligible participants were adults aged ≥19 years who met the diagnostic criteria for MOH based on the International Classification of Headache Disorders, 3rd edition (ICHD-3) [14]. Board-certified headache specialists at each center conducted structured interviews to confirm eligibility. Migraine diagnosis was determined at baseline by board-certified headache specialists through structured interviews based on the current ICHD-3 criteria. As this diagnosis reflects the clinical features at baseline inclusion, some patients may have experienced phenotype transformation (e.g., from tension-type headache to migraine-like MOH), leading to classification under migraine. Additional inclusion criteria included the ability to complete self-administered questionnaires and provide written informed consent. Patients with severe neurological, psychiatric, or medical conditions that could compromise study participation or data integrity were excluded.

For this secondary analysis, participants were stratified into two age groups: older (≥65 years) and younger (<65 years) groups. The threshold of 65 years is consistent with international epidemiological standards and is widely used in clinical and public health research [15]. It is consistent with definitions used by the World Health Organization and reflected in national policies such as Korea’s Act on Welfare of the Aged [16]. This cut-off has also been adopted in previous studies on aging-related headache disorders to assess age-specific differences in MOH presentation and treatment response [17,18].

### 2.2. Data Collection

Clinical data were collected from the RELEASE registry using standardized case report forms and structured questionnaires at baseline and follow-up. Data included demographics (age, sex, and body mass index (BMI)), lifestyle habits (smoking, alcohol, caffeine use), and medical comorbidities. Headache-related history encompassed age at headache onset, time to chronification, and duration of medication overuse.

Detailed information on acute and preventive medication use was collected. Acute medications were classified into six categories: ergotamines, triptans, simple analgesics (e.g., acetaminophen and non-steroidal anti-inflammatory drugs (NSAIDs)), combination analgesics, opioids, and others. Overuse type and frequency were recorded. Prescribed preventive treatments included both pharmacological and non-pharmacological modalities. Pharmacological therapies comprised antiepileptics (e.g., topiramate, valproate), beta-blockers (e.g., propranolol), calcium channel blockers (e.g., flunarizine, verapamil), tricyclic antidepressants (e.g., amitriptyline), serotonin–norepinephrine reuptake inhibitors, angiotensin-converting enzyme inhibitors or angiotensin II receptor blockers (e.g., candesartan), onabotulinumtoxinA, corticosteroids, calcitonin gene-related peptide (CGRP) monoclonal antibodies, and gepants. Interventional treatments included greater occipital nerve blocks, while non-pharmacological approaches included transcutaneous electrical nerve stimulation (e.g., Cefaly). Treatment strategy data included withdrawal method (abrupt, tapering, or continued use), treatment setting (inpatient or outpatient), and timing of preventive treatment initiation (none, early, or delayed). Medication use and treatment response were assessed at baseline and follow-up. Treatment response was defined as a ≥50% reduction in monthly headache days (MHD) from baseline to 3 months.

### 2.3. Clinical Assessments and Questionnaires

To evaluate headache impact, quality of life, psychological symptoms, and treatment satisfaction, participants completed a series of validated questionnaires in Korean at baseline and follow-up. Headache-related disability was assessed using the Migraine Disability Assessment (MIDAS) [19] and the Headache Impact Test-6 (HIT-6) [20]. Quality of life was evaluated using the EuroQol 5-Dimension [21] instrument and the Migraine-Specific Quality of Life Questionnaire version 2.1 [20]. Psychological status was assessed with the Patient Health Questionnaire-9 (PHQ-9) for depression, Generalized Anxiety Disorder-7 (GAD-7) for anxiety, and the Perceived Stress Scale short form (PSS-4) [22] for subjective stress levels. The presence of allodynia was measured using the Allodynia Symptom Checklist-12 (ASC-12), with a total score of ≥3 considered indicative of allodynia [23]. Patient satisfaction with acute treatment was assessed using the Migraine Assessment of Current Therapy (Migraine-ACT) questionnaire [24].

Assessments were conducted at baseline and repeated at 1, 3, 6, and 12 months. At each follow-up, data on MHD, acute medication use days, severe headache days, headache-free days, functional impairment, and healthcare utilization were collected. Although longitudinal data were collected for up to 12 months, the present study reports only the 3-month follow-up results.

### 2.4. Outcome Definition

The primary outcome was the proportion of patients achieving ≥50% reduction in MHD at 3 months compared to baseline (i.e., “treatment responders”). Adherence to preventive treatment over the 3-month period was classified into five categories based on the proportion of days with medication use: 100%, 75–99%, 50–74%, 25–49%, and ≤24%. Specifically, 100% adherence indicated continuous use (e.g., monthly CGRP monoclonal antibody injections or daily oral medications taken for at least 85 days). Partial adherence was further defined as follows: 75–99% for ≥6 weeks or ≥25 days/month, 50–74% for 4–5 weeks or 16–24 days/month, 25–49% for 2–3 weeks or 8–15 days/month, and ≤24% for minimal use (≤1 week or ≤7 days/month). Good adherence was defined as compliance of ≥50%, encompassing the top three adherence categories.

### 2.5. Statistical Analysis

All statistical analyses were performed using SPSS version 24.0. (IBM Corp., Armonk, NY, USA). Continuous variables were expressed as medians and interquartile ranges and compared using the Mann–Whitney U test. Categorical variables were summarized as counts and percentages and analyzed using the Chi-square test or Fisher’s exact test, as appropriate. A multivariate logistic regression analysis was conducted to identify predictors of treatment response (≥50% reduction in MHD) in older patients. Variables included demographic, clinical, and psychosocial factors. Odds ratios (ORs) and 95% confidence intervals (CIs) were reported. A *p*-value < 0.05 was considered statistically significant.

## 3. Results

### 3.1. Baseline Characteristics

Of the 791 patients enrolled in the RELEASE registry, 72 (9.1%) were classified as older (aged ≥ 65 years), and 719 (90.9%) as younger (aged < 65 years). As shown in Table 1, the proportion of female patients was slightly lower in the older group compared to the younger group (76.4% vs. 84.7%, *p* = 0.067). Older patients had a significantly higher median BMI (24.1 vs. 22.6 kg/m^2^, *p* = 0.005), and both headache onset and initiation of medication overuse occurred at significantly later ages (33.0 vs. 21.0 years and 61.5 vs. 39.0 years, respectively; both *p* < 0.001). Migraine was less frequently identified as the primary headache disorder in older patients than in younger counterparts (91.7% vs. 99.0%, *p* < 0.001).

**Figure 1:**
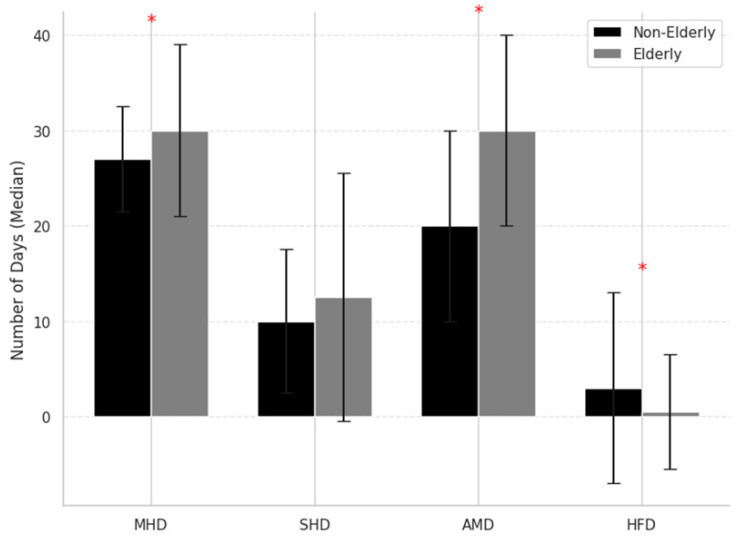
Comparison of headache characteristics between older and younger patients with medication overuse headache at baseline. Asterisks (*) indicate statistically significant differences between groups (*p* < 0.05).

Regarding headache profile, older patients reported significantly more MHD (30.0 vs. 27.0, *p* = 0.012), more days with acute medication use (30.0 vs. 20.0, *p* < 0.001), and fewer clear (headache-free) days (0.0 vs. 3.0, *p* = 0.012). They also experienced a more severe MHD (12.5 vs. 10.0, *p* = 0.056; Figure 1). Despite this, older patients had significantly lower disability scores, with reduced MIDAS scores (30.0 vs. 46.0, *p* < 0.001) and a trend toward lower HIT-6 scores (64.5 vs. 66.0, *p* = 0.065), as shown in Table 1.

Bar graphs depict median values and interquartile ranges for monthly headache days (MHD), monthly severe headache days (SHD), monthly acute medication use days (AMD), and clear (headache-free) days (HFD) in older (≥65 years) and younger (<65 years) patients. Older patients showed more frequent headaches and medication use, but fewer headache-free days. Asterisks (*) indicate statistically significant differences between groups (*p* < 0.05, Mann–Whitney U test). MHD: Monthly Headache Days; SHD: Severe Headache Days; AMD: Acute Medication Days; HFD: Headache-Free Days.

As summarized in Table 2, psychological measures (PHQ-9, GAD-7, and PSS-4) did not differ significantly between groups. However, older patients had significantly lower allodynia scores (ASC-12: 1.2 ± 2.2 vs. 2.2 ± 3.2, *p* = 0.001), greater satisfaction with acute treatment (ACT-4: 2.9 ± 1.4 vs. 2.4 ± 1.6, *p* = 0.002), and higher migraine-specific quality of life (MSQ total score: 196.4 ± 69.5 vs. 174.8 ± 68.7, *p* = 0.011). Older patients were more likely to overuse combination analgesics (48.6% vs. 25.6%, *p* < 0.001), and less likely to overuse triptan (23.6% vs. 41.7%, *p* = 0.003) or NSAIDs/simple analgesics (37.5% vs. 57.6%, *p* = 0.001). There were no significant differences in the use of opioids or ergotamines. Treatment approaches also differed: older patients were more likely to undergo inpatient withdrawal (19.4% vs. 11.7%, *p* = 0.057), and less likely to initiate preventive treatment at the time of withdrawal (81.9% vs. 92.4%, *p* = 0.003). A higher proportion of older patients received no preventive therapy (15.3% vs. 5.3%).

### 3.2. Preventive Treatment and 3-Month Outcomes

At 3-month follow-up, outcome data were available for 56 older and 569 younger patients. As shown in Table 3, overall adherence to preventive treatment was low in both groups, with no significant difference in the proportion of patients with good adherence (≥50%) (14.3% vs. 15.6%, *p* = 0.448). However, more older patients were prescribed but did not initiate preventive treatment (40.3% vs. 27.8%). Both groups showed marked improvement in headache-related outcomes. Older patients had a slightly greater reduction in MHD (8.5 vs. 10.0), and significantly more headache-free days (20.0 vs. 15.0, *p* = 0.001). However, they reported more monthly severe headache days compared to younger patients (3.5 vs. 2.0, *p* = 0.005). The proportion of treatment responders (≥50% reduction in MHD) was higher among older patients (50.0% vs. 41.6%), although the difference was not statistically significant (Table 3).

**Figure 2:**
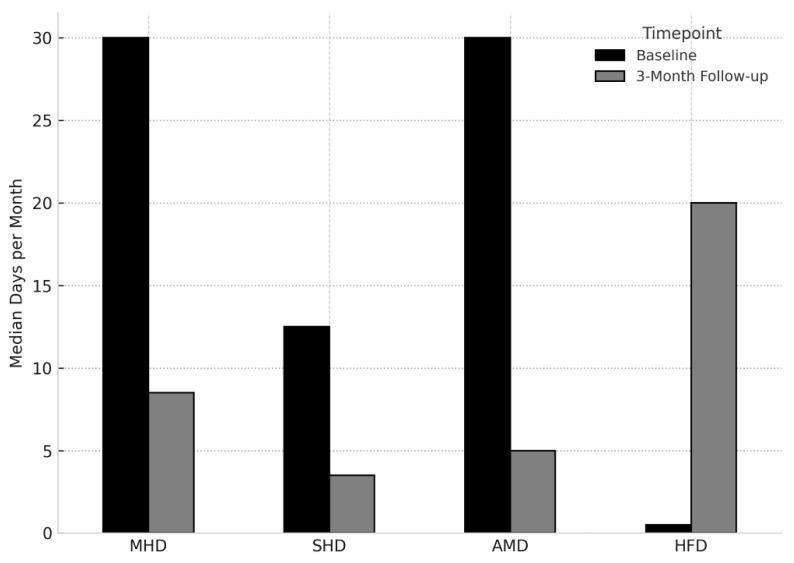
Comparison of headache-related outcomes from baseline to 3-month follow-up in older adults with medication overuse headache.

As shown in Figure 2, older adults with MOH exhibited significant improvements across multiple headache-related outcomes at the 3-month follow-up. MHD, severe headache days, and acute medication use days were significantly reduced compared to baseline, while the number of headache-free days increased, indicating a favorable response to treatment.

Bar plots show the mean ± standard error for monthly headache days (MHD), severe headache days (SHD), acute medication intake days (AMD), and headache-free days (HFD) at baseline and at 3-month follow-up in older adults (aged ≥ 65 years) with MOH. MHD: Monthly Headache Days; SHD: Severe Headache Days; AMD: Acute Medication Days; HFD: Headache-Free Days.

### 3.3. Predictors of Treatment Response in Older Patients

Multivariate logistic regression analysis revealed that most demographic, clinical and psychosocial variables—including sex, BMI, duration of medication overuse, MIDAS, HIT-6, PHQ-9, GAD-7, and ASC-12—were not significantly associated with treatment response. However, poor adherence to preventive therapy (≤24%) was significantly associated with a reduced likelihood of treatment response (OR = 0.13, 95% CI: 0.02–0.96, *p* = 0.045), as detailed in Table 4.

### 3.4. Overused Medication Type and Response

Among older patients, no statistically significant association was observed between treatment response and the type of overused acute medication. OR for triptans, simple analgesics/NSAIDs, combination analgesics, ergotamine, and opioids showed wide confidence intervals and non-significant *p*-values, indicating high variability (Table 5). Although numerically lower response rates were observed in patients overusing triptans, combination analgesics, or simple analgesics/NSAIDs, these differences did not reach statistical significance.

## 4. Discussion

This study demonstrates that older adults with MOH exhibit distinct clinical characteristics compared to younger adults. Despite experiencing more frequent headaches and higher use of acute medications, they reported lower levels of disability and greater satisfaction with acute treatment. These findings are consistent with prior studies showing that older adults often report fewer associated symptoms (e.g., nausea, photophobia, phonophobia) and underutilize preventive treatments despite comparable headache frequency [25,26,27]. Although migraine prevalence tends to decrease with age [28], over 90% of older patients in our cohort had comorbid migraine, suggesting that MOH frequently arises on a migraine background in this population.

Preventive medications were prescribed in both groups, but patterns of use and adherence differed. Older adults were less likely to initiate certain agents—particularly beta-blockers (8.3% vs. 20.0%) and CGRP monoclonal antibodies (15.3% vs. 25.5%, Table 2)—and were more likely to decline or discontinue prescribed preventive treatments. Good adherence (≥50%) was comparably low in both groups (14.3% vs. 15.6%), but more older adults did not take prescribed treatments at all (40.3% vs. 27.8%, Table 3). This may reflect concerns related to polypharmacy, comorbidities, and drug tolerability, which are more common in older adults due to altered pharmacokinetics and increased sensitivity to side effects [9,28,29].

The exceptionally high baseline MHD in both age groups—30 days/month in older adults and 27 days/month in younger adults—likely reflects the inclusion criteria of our registry, which required a diagnosis of MOH. This condition is typically associated with higher headache frequency than chronic migraine alone, often exceeding 25 days per month [30,31]. Moreover, as the RELEASE registry is based in tertiary headache centers, referral bias may have led to the inclusion of more severely affected or treatment-refractory patients [13,32].

Age-related differences in clinical presentation and treatment response were evident. Older patients more frequently overused combination analgesics and were less likely to use triptans or NSAIDs, possibly due to physician caution or contraindications with underlying comorbidities [33,34]. Interestingly, despite higher headache frequency, older patients reported lower MIDAS scores. This dissociation may result from age-related differences in symptom perception and functional expectations, including the tendency to underreport pain or prioritize daily functioning over complete symptom relief [7,27,35,36,37,38]. In line with this, older adults exhibited lower allodynia scores and higher acute treatment satisfaction despite similar levels of psychological distress [39,40].

Very poor adherence (≤24%) was independently associated with reduced treatment response, emphasizing the importance of compliance. Although treatment response did not differ significantly by the type of overused medication, patients overusing triptans, combination analgesics, or NSAIDs showed numerically lower response rates. These trends, though not statistically significant, may reflect underlying heterogeneity in headache subtypes and warrant further investigation [41]. Importantly, older adults with good adherence still experienced favorable outcomes, including a 50% response rate at 3 months. This finding underscores the importance of promoting adherence in older adults, who can benefit significantly from preventive therapy when properly followed.

Given the challenges of polypharmacy and adverse effect risks, detoxification alone—without concomitant preventive therapy—may be a practical and effective strategy for selected older adults with low disability or strong motivation for behavioral change [42,43]. This approach could reduce the treatment burden while still achieving meaningful clinical improvement. Tailored management strategies that prioritize safety, tolerability, and patient preferences are essential in this population.

These findings reinforce the need for age-specific MOH management strategies, as previously highlighted in older headache literature [8,26,44]. Effective age-specific approaches should include (1) careful assessment of comorbidities and polypharmacy to guide medication choices, (2) patient-centered education to improve long-term adherence, and (3) close monitoring to detect early signs of treatment non-response or intolerance. Additional practical considerations include conducting medication reconciliation to reduce the burden of polypharmacy, initiating simplified and well-tolerated regimens, and considering detoxification without preventive therapy for selected patients with lower disability. Tailored follow-up and behavioral support may further improve treatment engagement in this population. These strategies may help optimize outcomes while minimizing adverse effects in this vulnerable population.

Moreover, the pharmacological vulnerability of older adults—due to altered pharmacokinetics, polypharmacy, and increased sensitivity to side effects—should be carefully considered when initiating or adjusting treatments. For some patients, detoxification without preventive therapy may be a viable and effective approach, especially in those with lower disability levels or higher engagement in behavioral modification. Our findings support the need for individualized treatment strategies that prioritize safety, tolerability, and patient preferences in older adults with MOH. Polypharmacy, typically defined as the use of five or more concurrent medications, is common among older adults and presents a challenge for headache management. In the context of MOH, multiple comorbid conditions often require pharmacologic treatment, increasing the risk of drug–drug interactions, side effects, and poor tolerability of additional preventive medications. This complex medication burden may contribute to the lower adherence observed in our older cohort.

This study has several limitations. First, while it was based on a prospective registry, causal relationships cannot be confirmed. Second, long-term outcome data were not available, limiting insight into recurrence and prognosis. Third, the relatively small number of older adults (9.1%) reflects the lower real-world prevalence of MOH in this age group and the tertiary care setting, which may reduce statistical power for subgroup analyses. Additionally, although gender may influence headache characteristics and treatment response, sex-stratified analyses were not feasible. Accurate quantification of failed preventive treatment lines was also limited by heterogeneity in medication types, dosages, treatment durations, and reasons for discontinuation. This constrained the ability to assess treatment resistance systematically. Non-headache medications were not systematically recorded, precluding formal analysis of polypharmacy and its impact on adherence and tolerability. While the proportions of prescribed medications were available, adherence to individual preventive agents could not be reliably assessed due to variability in treatment duration, dosage, and patient-reported compliance. This limited our ability to examine drug-specific adherence patterns or their impact on outcomes. Finally, the use of MIDAS and HIT-6 to assess headache-related disability may have introduced bias, particularly in older adults. These tools include work-related items that may not apply to retired individuals, potentially underestimating disability in this demographic.

## 5. Conclusions

Older patients with MOH demonstrated distinct clinical features, including more frequent headaches and acute medication use, lower preventive treatment rates, and greater treatment satisfaction, despite increased headache frequency. Notably, they also exhibited lower disability scores and better quality of life compared to younger patients. These results highlight the importance of age-sensitive approaches to the diagnosis, management, and follow-up of MOH. Improving adherence to preventive therapies should be a particular focus in older populations. Further longitudinal research is needed to clarify the clinical trajectory, care barriers, and optimal treatment strategies for older patients with MOH.

## Figures and Tables

**Table 1:** Comparison of clinical features between older and younger patients with medication overused headache (N = 791).

Characteristics	Older Adult <65 Years (N = 719)	Younger Adult ≥65 Years (N = 72)	*p*-Value
Demographics			
Sex, female, *n* (%)	609 (84.7)	55 (76.4)	0.067
BMI, kg/m^2^	22.6 (20.3, 25.2)	24.1 (21.9, 26.0)	0.005 *
Headache History			
Age at headache onset, years	21.0 (16.0, 30.0)	33.0 (24.0, 51.0)	<0.001 *
Age at medication overuse onset, years	39.0 (31.0, 48.0)	61.5 (52.0, 67.5)	<0.001 *
Migraine diagnosis, *n* (%)	712 (99.0)	66 (91.7)	<0.001 *
Types of Overused Medication			
Ergotamine, *n* (%)	115 (16.0)	7 (9.7)	0.160
Triptans, *n* (%)	300 (41.7)	17 (23.6)	0.003 *
Simple analgesics/NSAIDs, *n* (%)	414 (57.6)	27 (37.5)	0.001 *
Opioids, *n* (%)	21 (2.9)	3 (4.2)	0.557
Combination analgesics, *n* (%)	184 (25.6)	35 (48.6)	<0.001 *
Headache Profile at Baseline			
Monthly headache days, days/month	27.0 (20.0, 30.0)	30.0 (21.0, 30.0)	0.012 *
Monthly severe headache days, days/month	10.0 (5.0, 15.0)	12.5 (7.0, 20.0)	0.056
Monthly acute medication days, days/month	20.0 (15.0, 30.0)	30.0 (20.0, 30.0)	<0.001 *
Clear (headache-free) days, days/month	3.0 (0.0, 10.0)	0.0 (0.0, 9.0)	0.012 *
HIT-6 score	66.0 (62.0, 70.0)	64.5 (60.0, 70.0)	0.065
MIDAS	46.0 (20.0, 100.0)	30.0 (0.0, 65.0)	<0.001 *

Data are presented as medians with interquartile ranges for continuous variables and as counts with percentages for categorical variables, as appropriate. Continuous variables were compared using the Mann–Whitney U test, and categorical variables were analyzed using the Chi-square test or Fisher’s exact test, depending on expected frequencies. A two-tailed *p*-value < 0.05 was considered statistically significant. Statistically significant values are marked with an asterisk (*).

**Table 2:** Psychosocial measures and treatment characteristics in older vs. younger adult medication overused headache patients.

Characteristics	Younger Adult <65 Years (N = 719)	Older Adult ≥65 Years (N = 72)	*p*-Value
Psychological and Functional Status at Baseline			
PHQ-9	9.0 (6.0, 15.0)	11.0 (5.5, 17.0)	0.334
GAD-7	6.0 (2.0, 10.0)	7.0 (2.5, 12.0)	0.267
ASC-12	1.0 (0.0, 4.0)	0.0 (0.0, 1.5)	0.005 *
PSS-4	8.0 (6.0, 10.0)	8.0 (7.0, 10.0)	0.465
ACT-4	3.0 (1.0, 4.0)	4.0 (2.0, 4.0)	0.003 *
MSQ total score	183.3 (128.1, 227.1)	207.6 (153.8, 249.6)	0.010 *
Types of Preventive Treatment			
Antiepileptics	367 (51.0)	33 (45.8)	
Beta-blockers	144 (20.0)	6 (8.3)	
Calcium channel blockers	95 (13.2)	11 (15.3)	
Tricyclic antidepressants	200 (27.8)	16 (22.2)	
Angiotensin receptor blockers	17 (2.4)	0 (0.0)	
Serotonin–norepinephrine reuptake blockers	11 (1.5)	1 (1.4)	
OnabotulinumtoxinA (BOTOX)	80 (11.1)	8 (11.1)	
Steroid	5 (7.0)	0 (0.0)	
CGRP mAb	183 (25.5)	11 (15.3)	
Gepants	1 (0.1)	0 (0.0)	
Cefaly	1 (0.1)	0 (0.0)	
GONB	26 (3.6)	1 (1.4)	
Treatment Strategies			
Mode of withdrawal			0.557
Abrupt discontinuation, *n* (%)	359 (49.9)	39 (54.2)	
Reduced frequency, *n* (%)	323 (44.9)	28 (38.9)	
No withdrawal, *n* (%)	37 (5.6)	5 (6.9)	
Setting of withdrawal (admission), *n* (%)	84 (11.7)	14 (19.4)	0.057
Preventive treatment use			0.003 *
None, *n* (%)	38 (5.3)	11 (15.3)	
Early initiation (from withdrawal), *n* (%)	664 (92.4)	59 (81.9)	
Late initiation (>2 weeks), *n* (%)	17 (2.4)	2 (2.8)	

Data are presented as medians with interquartile ranges for continuous variables and as counts with percentages for categorical variables, as appropriate. Continuous variables were compared using the Mann–Whitney U test, and categorical variables were analyzed using the Chi-square test or Fisher’s exact test, depending on expected frequencies. A two-tailed *p*-value < 0.05 was considered statistically significant. Statistically significant values are marked with an asterisk (*).

**Table 3:** Preventive treatment and 3-month headache outcomes (3-month follow-up: Younger N = 569, Older N = 56).

Characteristics	Younger Adult <65 Years (N = 569)	Older Adult ≥65 Years (N = 56)	*p*-Value
Preventive Treatment Compliance			0.448
100%	63 (8.8)	6 (8.3)	
75~99% (≥6 weeks or ≥25 days/month)	13 (1.8)	1 (1.4)	
50~74% (4~5 weeks or 16~24 days/month)	13 (1.8)	1 (1.4)	
25~49% (2~3 weeks or 8~15 days/month)	18 (2.5)	2 (2.8)	
≤24% (≤1 weeks or ≤7 days/month)	41 (5.7)	3 (4.2)	
Prescribed but not taken	200 (27.8)	29 (40.3)	
Not prescribed	221 (30.7)	14 (19.4)	
Good compliance (≥50%), *n* (%)	89 (15.6)	8 (14.3)	
Headache Outcomes			
Monthly headache days, days/month	10.0 (1.0, 20.0)	8.5 (3.5, 15.0)	0.662
Monthly severe headache days, days/month	2.0 (0.0, 6.0)	3.5 (1.0, 7.0)	0.005 *
Monthly acute medication days, days/month	5.0 (0.0, 11.0)	5.0 (2.0, 10.0)	0.926
Clear (headache-free) days, days/month	15.0 (0.0, 22.0)	20.0 (10.0, 24.5)	0.001 *
HIT-6 score	45.0 (0.0, 58.0)	50.0 (0.0, 57.5)	0.371
MIDAS	0.0 (0.0, 20.0)	2.5 (0.0, 20.0)	0.242
Responder (≥50% reduction in MHD), *n* (%)	299 (41.6)	36 (50.0)	

Data are presented as medians with interquartile ranges or counts with percentages, as appropriate. Continuous variables were compared using the Mann–Whitney U test, while categorical variables were analyzed using the Chi-square test or Fisher’s exact test, depending on expected frequencies. A two-tailed *p*-value < 0.05 was considered statistically significant. Statistically significant values are marked with an asterisk (*).

**Table 4:** Predictors of treatment response (≥50% reduction in MHD) in older patients with medication overuse headache.

Predictor	OR	95%CI	*p*-Value
Female	1.39	(0.32–6.08)	0.665
BMI	1.02	(0.83–1.25)	0.881
Duration from medication overuse onset	1.04	(0.98–1.10)	0.216
Duration from headache onset	1.00	(0.96–1.04)	0.935
HIT-6	1.01	(0.93–1.09)	0.879
MIDAS	1.00	(0.98–1.01)	0.486
PHQ-9	0.93	(0.79–1.08)	0.344
GAD-7	1.05	(0.88–1.26)	0.583
ASC-12	1.23	(0.86–1.76)	0.253
PSS-4	1.08	(0.77–1.51)	0.652
ACT-4	1.07	(0.67–1.70)	0.786
Total MSQ	1.00	(0.99–1.02)	0.505
Compliance <24%	0.13	(0.02–0.96)	0.045
Abrupt withdrawal	1.15	(0.07–18.20)	0.920
Early Preventive drug start	7.81	(0.14–437.36)	0.317

Multivariate logistic regression analysis was conducted to identify predictors of treatment response among older patients with MOH. Among various clinical, psychosocial, and treatment-related variables, poor compliance to preventive treatment (defined as ≤24% adherence) was significantly associated with non-response. Other factors were not statistically significant.

**Table 5:** Association between overused medication type and treatment response in older patients with MOH.

Medication Type	OR	95%CI	*p*-Value
Ergotamine	1.38	(0.28–6.64)	1.000
Triptans	0.86	(0.29–2.55)	1.000
Simple analgesics/NSAIDs	1.43	(0.55–3.73)	0.627
Opioids	2.06	(0.18–23.77)	1.000
Combination analgesics	1.40	(0.55–3.53)	0.638

Logistic regression analysis was used to examine the relationship between the type of overused acute medication and treatment response in older patients. No statistically significant associations were observed across medication classes.

## Data Availability

The data supporting the findings of this study are available from the corresponding author upon reasonable request.
